# Seasonal Malaria Chemoprevention: An Evolving Research Paradigm

**DOI:** 10.1371/journal.pmed.1002176

**Published:** 2016-11-22

**Authors:** Robert W. Snow

**Affiliations:** 1 KEMRI-Wellcome Trust Collaborative Programme, Nairobi, Kenya; 2 Centre for Tropical Medicine and Global Health, Nuffield Department of Clinical Medicine, University of Oxford, Oxford, United Kingdom

## Abstract

Robert W. Snow discusses the importance of empirical evidence, such as that provided in the trial published this week by Milligan and colleagues, in guiding malaria control in Africa.

In this issue, Paul Milligan and colleagues [1] provide evidence of the dramatic impact on the incidence of malaria of seasonal malaria chemoprevention (SMC) among children aged older than five years, the current recommended upper age limit for SMC. Research on drugs to prevent malaria in Africa among nonpregnant populations has a long history with surprisingly little sustainable policy impact. The last five years of SMC research signal an important stage in drug-based policy for malaria based on evolving field science.

## Antimalarial Drugs to Prevent Malaria in Africa

The use of antimalarial drugs to prevent malaria in Africa is not new [2,3]. At the turn of the last century, quininisation was widely promoted among residents of colonial administrative centres. During the “eradication” projects of the 1950s and 1960s, when indoor residual spraying failed to reach expected targets, mass drug administration (MDA) was included, resulting in huge reductions in infection incidence but never quite reaching elimination. The seasonal use of “chemoprophylaxis” was undertaken as part of pilot trials or elimination campaigns in Kenya, Burkina Faso, Senegal, Reunion, and Tunisia, again with dramatic impacts on parasite transmission and disease incidence [2,3]. Approaches to the seasonal use of drugs to prevent infection were resurrected during the 1980s with trials of fortnightly distribution by village health workers of pyrimethamine-dapsone to young children in The Gambia, resulting in an 80% reduction in clinical events and a 34% reduction in all-cause childhood mortality [4]. Trials during the early 2000s of intermittent presumptive sulphadoxine-pyrimethamine (SP) treatment of infants (IPTi) attending routine vaccine visits showed on average a 30% protection against morbid events due to malaria [5]. However, with the exception of the presumptive SP treatment of malaria in pregnancy, throughout the history of malaria control in Africa, drug-based interventions have received less attention compared to vector control. All commentators, since the earliest use of quinine and chloroquine prophylaxis, have highlighted concerns related to adherence, adequate coverage, sustainability, delayed acquisition of immunity, and resistance, concerns relevant to both vector- and drug-based control.

## Seasonal Malaria Chemoprevention (SMC)

Trial results of SMC, between 2006 and 2011, showed dramatic impacts of the intermittent, presumptive use of combinations of antimalarial drugs on the incidence of clinical malaria in children aged between two months and five years [6]. A meta-analysis in 2012 found strong evidence that the periodic, presumptive use of SP in combination with Amodiaquine (AQ) in areas of acute seasonal transmission could reduce malaria morbidity in young children by 75% [6]. This evidence led to policy statements by WHO the same year [7] and development of regional and national plans for implementation of SMC. Donor agencies provided funding to operational plans through consortia of national malaria control programmes, nongovernmental organisations, UN agencies, and monitoring and evaluation partners. Within a year, 3.2 million children aged less than five years were protected by SMC in seven countries [8]. This history provides an exemplary illustration of how field research evidence can lead to early policy adoption and immediate donor assistance. Importantly, previous reservations on the use of drugs for malaria control seemed less of a concern for SMC than, say, for IPTi or MDA.

## Why Restrict SMC to Children Aged Less Than Five Years?

**Fig 1:**
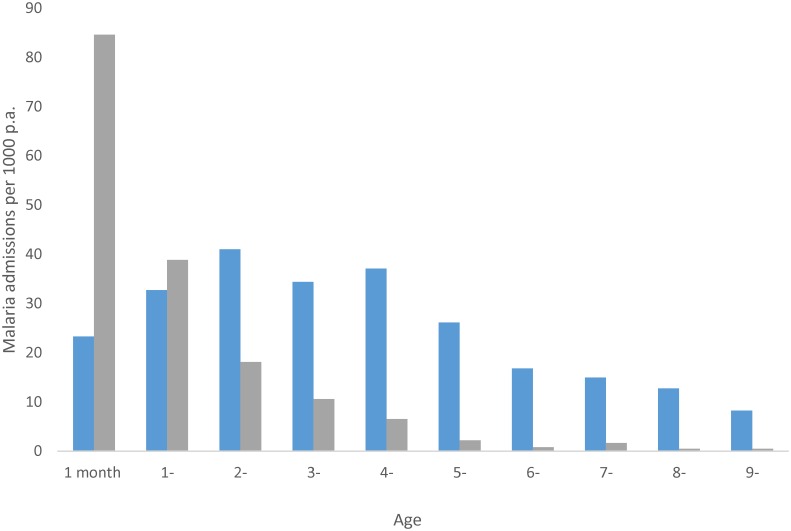
Annual age-specific rates of malaria admissions to hospital from communities where the prevalence of infection is circa 75% (grey bars) and circa 30% (blue bars); *x*-axis starts at 1–11 months of age and year since last birthday thereafter; adapted from [9].

The operational costs of reaching households with children under the age of five would be similar if one aimed to reach these children’s older siblings at the same time. The only additional costs would be the increased use of comparatively cheap, well-tolerated drug combinations, while the benefits could be great if disease burdens were significant in children above five years of age. The epidemiological associations among parasite exposure, age, and clinical burden are complex, but, in broad terms, as malaria transmission intensity declines, the age at which functional clinical immunity is acquired increases (Fig 1) [9,10]. West Africa and the Sahel, where current SMC efforts, including Milligan and colleagues’ trial, are focussed, encompass a wide range of intrinsic transmission characteristics, important to predict the impact of SMC [11]. Similarly varied has been the ability to reduce transmission potential through vector control. Countries such as Senegal, where Milligan and colleagues conducted their trial, and The Gambia have witnessed massive reductions in malaria transmission intensity over the last decade, to the extent that the phenotype of clinical malaria has transitioned from a disease concentrated in young children to one that affects an older childhood population [12,13].

Milligan and colleagues used a step-wedged design to incrementally test the effects of monthly distribution of SP+AQ to children under the age of ten years during the short malaria season in Senegal [1]. They found a 60% reduction in malaria incidence [1]. This empirical evidence demonstrates the benefits that can be gained by increasing the age window of targeted SMC in areas where transmission intensity is either already moderately low or brought down by vector control. Reducing the reservoir of infection will also have a wider impact on transmission. When SMC coverage is as high as 80% and insecticide-treated net (ITN) use is also high, these combined interventions will continue to change the epidemiology of parasite exposure and the clinical landscape.

## Sustaining a Dynamic Research Agenda

Parasites, vectors, and humans adapt in the face of intervention. Models might be able to predict what might happen, but they do not tell you what does happen. We have become too comfortable with model predictions of impact and future predictions. After 20 years of scaling access to ITN, we still depend on models on their likely contribution to changing disease burdens. Empirical evidence is scanty, the impact of ITN on the disease phenotype and acquired immunity is poorly described, and pyrethroid resistance has emerged, but its public health impact remains unclear.

Among evidence-based policy developments, SMC is notable for incorporating the idea that different approaches may be appropriate in different settings. Maintaining careful epidemiologic surveillance alongside sustained and expanding intervention coverage should become a requirement for national malaria control programmes to redefine target populations and ensure there is no rebound [14]. New drugs are needed, and the Medicines for Malaria Venture has already included ideal target profiles for SMC drugs in their product development portfolio [15]. Operational research on how to access those most distant from regular health services is urgently needed. Measuring how many malaria events are prevented, without relying solely upon theoretical models, will provide the incentive for continued donor assistance. Even if a fraction of the costs of delivering SMC were dedicated to intensive surveillance and a sustained research agenda, national malaria programmes could adapt to a changing epidemiology, parasite resistance, and community acceptability. Importantly, ten years from now we won’t be left guessing whether this intervention is still working.

## Abbreviations

AQ: Amodiaquine
IPTi: intermittent presumptive treatment of infants
ITN: insecticide-treated net
MDA: mass drug administration
SMC: seasonal malaria chemoprevention
SP: sulphadoxine-pyrimethamine
